# Pure Milk

**Published:** 1870-01

**Authors:** 


### PURE MILK.

We noticed, a year ago, in St. James' Park, London, that in order to insure pure milk for children, a place was set apart for cows, and when milk was wanted it was milked into a cup and drank by the children. We need not take that trouble in New York City, for pure milk is obtained at No. 71 Fourth avenue, 411 Seventh avenue, 1,331 Broadway, and 22 Boerum street, Brooklyn, furnished by the Rockland County Milk Association, of which S. W. Canfield, Esq., is President; Condensed Milk is also supplied at the same places. Milk from the same cow, or cream, will also be supplied every day for those who desire it for their children or for invalids. Our subscribers would do well to make a note of this.

